# Anatomical, histological and computed tomography comparisons of the eye and adnexa of crab-eating fox (*Cerdocyon thous*) to domestic dogs

**DOI:** 10.1371/journal.pone.0224245

**Published:** 2019-10-23

**Authors:** Nayone Lima Lantyer-Araujo, Danielle Nascimento Silva, Alessandra Estrela-Lima, Caterina Muramoto, Fernanda de Azevedo Libório, Érica Augusta da Silva, Arianne Pontes Oriá

**Affiliations:** 1 Post-Graduate Program in Animal Science in the Tropics, School of Veterinary Medicine and Zootechny, Federal University of Bahia (UFBA), Salvador, Bahia, Brazil; 2 Department of Veterinary Anatomy, Pathology and Clinics, School of Veterinary Medicine and Zootechny, UFBA, Salvador, Bahia, Brazil; 3 Screening Center for Wild Animals, Brazilian Institute of the Environment and Renewable Natural Resources, Salvador, Bahia, Brazil; University of Western Australia, AUSTRALIA

## Abstract

An understanding of species' morphological and physiological parameters is crucial to developing conservation strategies for wild animals kept in human care. Detailed information is lacking for crab-eating fox (*Cerdocyon thous*) eyes and adnexa. Therefore, the aim of this study was to describe anatomical, histological and computed tomography (CT) features of the eye and adnexa in crab-eating fox, compared to domestic dogs. CT of the eye and adnexa of one live animal and a frozen specimen was performed for anatomical identification. In addition, the heads of five animals of each species were fixed in 10% buffered formalin for gross anatomical description of the eye and adnexa using topographic dissection and exenteration techniques. All steps were photographed and features such as location, shape, and distances and relationships between structures were described. For histological evaluation, two eyes of each species were fixed in 10% buffered formalin, processed by routine paraffin inclusion technique and stained with hematoxylin and eosin. The CT scan was difficult to evaluate, mainly that of the frozen head, which did not provide good definition of the soft tissues; nevertheless, it demonstrated the potential for structure visualization and description. The gross anatomical and histological evaluations showed the presence of eyelashes on the upper eyelid and of upper and lower lacrimal points, an incomplete orbit with supraorbital ligament, slightly exposed sclera with discretely pigmented limbus and pigmentation throughout the conjunctiva, and a slit-shaped pupil. Hematoxylin and eosin staining demonstrated structural similarities between the crab-eating fox and domestic dog. Thus, the possibility of using the domestic dog as a study model for the preventive and therapeutic management of wild dogs kept in human care is demonstrated.

## Introduction

The crab-eating fox (*Cerdocyon thous*) is considered the most common canid in South America, widely distributed from Colombia to northern Argentina and a large part of Brazil [1, 2]. This species occupies most habitats in South America, including cerrado, caatinga, savannas, and Atlantic and Araucaria forests [3]. The Convention on International Trade in Endangered Species of Wild Fauna and Flora (CITES) has classified the crab-eating fox in Appendix II [4], and an extinction risk assessment carried out by the Chico Mendes Institute for Biodiversity Conservation (ICMBio) has classified it as of “Least Concern” due to its wide geographic distribution, great tolerance to anthropogenic disturbances and population stability [5]. A similar assessment was performed for the Red List of Threatened Species of The International Union for Conservation of Nature (IUCN) [3], once there were no precise estimates of population size.

The major risk to this species' conservation is related to the transmission of infectious diseases, often the result of approaching domestic dogs [3, 6]. Both species belong the same subfamily Caninae, but are positioned in different clades of the phylogenetic tree: the domestic dog into the wolf-like and the crab-eating fox into the South American clades [7]. Nevertheless, there are a number of infectious diseases, some of them zoonosis, in which the crab-eating fox can play an important role as natural reservoir [8]. The role of this species in the epidemiology of agents, such as *Erlichia*, *Hepatozoon*, *Toxoplasma gondii* and *Leishmania*, is largely studied, to promote proper monitoring in regions where they approach domestic animals and humans [8–10].

The clinical manifestations of such diseases in wild canids can differ from those in domestic dogs, with the former presenting mucopurulent ocular discharge, conjunctivitis or uveitis [8, 11, 12]. These animals therefore require ophthalmic evaluation and an accurate diagnosis, even if such an evaluation is challenging due to equipment that is either difficult to use or structurally challenging, and the need for animal restraint [13].

Studies on the anatomical and histological particularities of wild canids and those that correlate findings with computed tomography (CT) images are scarce. Among the species of carnivores, a gross study of morphometric features of the lacrimal and third eyelid glands was developed in domestic dogs, providing information for the formulation of therapeutic protocols and surgical strategies [14]. However, similar data that could aid in the management and treatment of crab-eating foxes are not available.

Diagnosis of ocular abnormalities, even of infectious, traumatic, metabolic or iatrogenic causes, requires specific knowledge of the ocular structure, the established ophthalmic parameters for the species, and available complementary examinations [15]. Knowledge of the anatomical and microscopic features of the visual system can serve to increase the preventive, diagnostic and therapeutic management of ocular diseases for these animals, whether they are kept in human care or rescued, and they can guide preventive or sanitary management. Thus, the objective of this study was to provide an anatomical, histological and CT description of the eye and adnexa of crab-eating fox, and compare this to findings in domestic dogs.

## Materials and methods

The research protocols were approved by the Ethics Committee for the Use of Experimental Animals of the School of Veterinary Medicine and Zootechny, Federal University of Bahia (protocol no. 73/2016) and are in accordance with the Authorization and Information System of Biodiversity, Brazilian Ministry of the Environment–SISBIO (process no. 27489–1). In addition, they were conducted according to the bioethics guidelines stated by the Association for Research in Vision and Ophthalmology (ARVO).

### Animals

One adult male crab-eating fox, weighing 5.5 kg, from the Triage Center of Wild Animals (CETAS–IBAMA–Salvador/BA) was evaluated by CT. In addition, for the CT, anatomical and histological studies, the heads of five adult crab-eating foxes, three female and two male, weighing between 5.1 and 5.6 kg were provided by CETAS–IBAMA–Salvador/BA and by the Department of Anatomy of the School of Veterinary Medicine and Zootechny (EMEVZ/UFBA), after the animals' death by natural causes, with no compromised bone or sense organs. For comparative assessment, five heads of adult mongrel mesaticephalic domestic dogs (*Canis lupus familiaris*), three female and two male, weighing between 4.2 and 8.6 kg, were obtained from the Laboratory of Veterinary Pathology (LPV/UFBA) and the Laboratory of Veterinary Pathology, Veterinary Medicine Hospital–UNIME. These animals died from other causes unrelated to this study and postmortem evaluation of the eye and adnexa were performed to verify the absence of any gross abnormalities.

### CT evaluation

For the CT exam, the animal was subjected to an intramuscularly dissociative anesthesia protocol using 5 mg/kg ketamine (Francotar^®^; Virbac Animal Health, São Paulo, Brazil) and 0.5 mg/kg midazolam (Dormire^®^; Cristália Produtos Químicos Farmacêuticos Ltda., São Paulo, Brazil). After 10 min, cephalic vein catheterization with a 22G catheter was performed to initiate general anesthesia using 5 mg/kg propofol, which was maintained, after intubation, with isoflurane (Isofluorano®, Biochimico, Rio de Janeiro/RJ, Brazil) administered by universal vaporizer.

The study was performed in the rostrocaudal direction, with the animal kept in ventral recumbence. Images were obtained in the axial plane using a CT helical scanner (Asteion^TM^ TSX-021B®, 4-detector row, Toshiba Medical Systems Corporation, Tochigi, Japan), with a rotation time of 1.0 s, voltage of 120 kVp, amperage of 150 mAs and slice thickness of 1.0 mm. Pre- and postcontrast scans were performed on the live animal and a noncontrast study on one frozen head. Iodinated contrast medium (Ioversol, Optiray 320®, Mallinckrodt Inc, EUA) was intravenously injected at a dose of 2.0 mL/kg. Images were reconstructed and exhibited with bone and soft tissue filters by Horos® medical image viewer. The objective of this evaluation was to identify structures in the live animal and in a cadaver, for macroscopic and morphometric evaluation of the eye and adnexa, and to aid in the identification of structures that are macroscopically difficult to visualize. After tomographic examination, the live animal was kept in an individual cage until full recovery and returned to his enclosure in the CETAS–IBAMA.

### Anatomical description of the eye and adnexa

We performed an anatomical study of the eye and adnexa on four crab-eating fox heads compared to five heads of adult domestic dogs at the LPV/UFBA. After identification of the animals, the atlanto-occipital joint was disarticulated to remove the head; 0.1 mL of 10% buffered formalin was injected (pH 7.3) in the medial and lateral region of the palpebral conjunctiva, and 0.2 mL in the posterior segment of the eye, for fixation of the internal structures. The heads were immersed in 10% phosphate buffered formalin at 10-fold the volume of the examined structures and macroscopically evaluated 48 h later.

Macroscopic evaluation of the visual system was performed using a topographic dissection and exenteration method, with a digital caliper (Mitutoyo, São Paulo, Brazil). A sagittal skin section from the frontal to nasal region of the head was performed and the skin was folded to expose retrobulbar structures. All steps were photographed with a camera with HD magnification lens and flash system (Nikon® D7000, Nikon Inc. Tokyo, Japan). Structure, location, shape, and distances and relationships between structures were described. After identification of external structures, the eye was sectioned in the dorsoventral plane, including the optic nerve, to identify the internal structures.

### Anatomical description of the orbit

**Fig 1:**
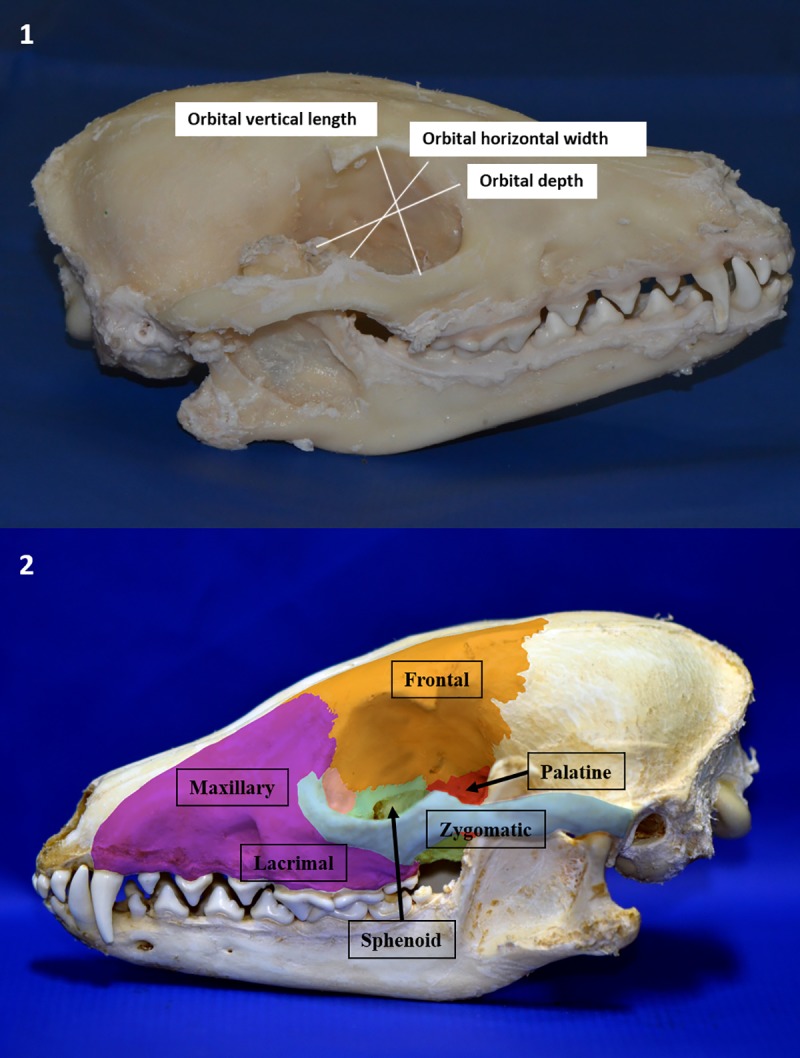
Image of macerated crab-eating fox head. (A1) Orbital vertical length. (B1) Orbital horizontal width. (C1) Orbital depth. Description of orbital bones: (a2) Frontal, (b2) maxillary, (c2) lacrimal, (d2) zygomatic, (e2) sphenoid and (f2) palatine.

**Fig 2:**
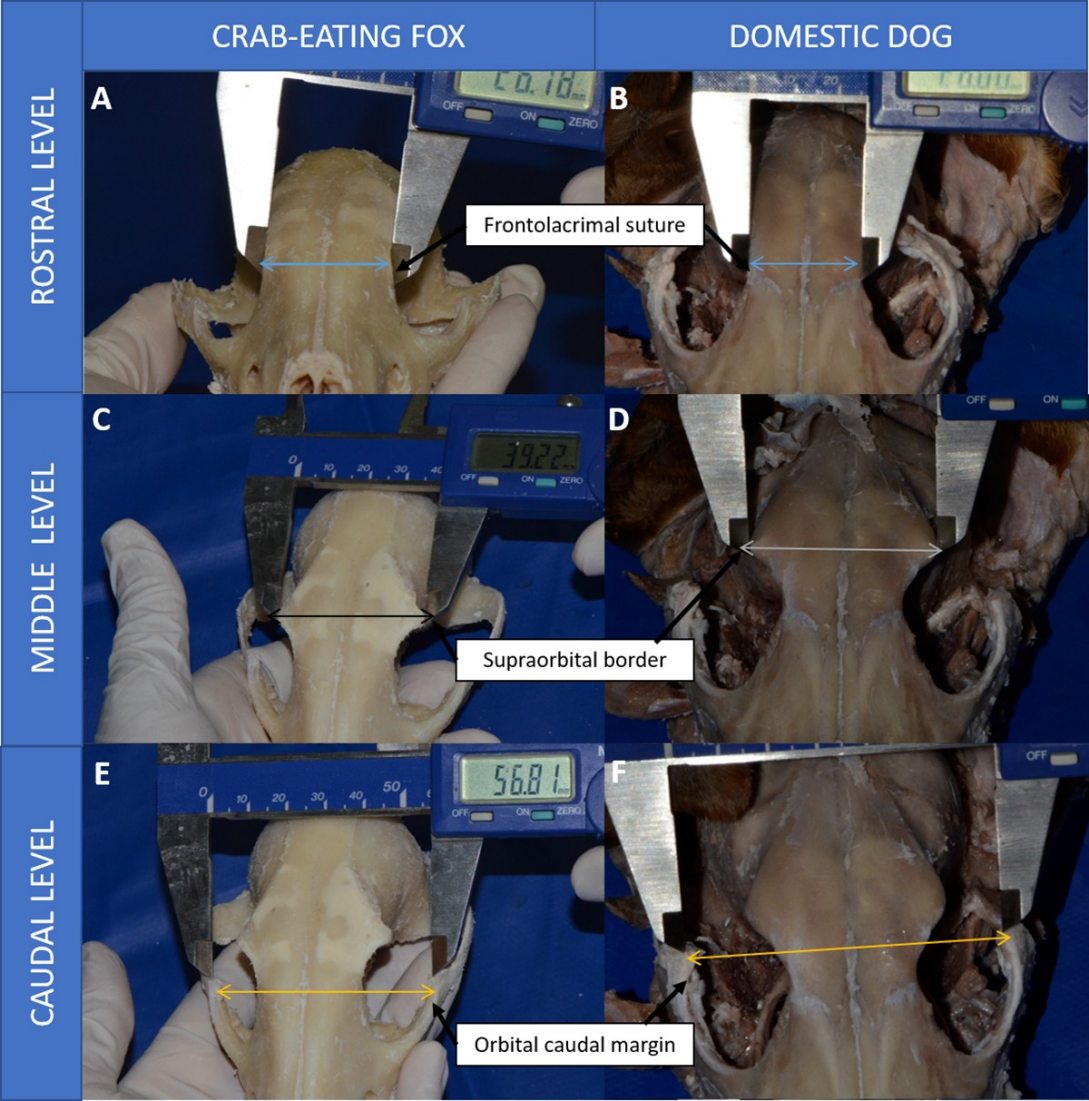
Image of macerated crab-eating fox head describing interorbital distances. (A) Rostral level, (C) middle level, (E) caudal level, in comparison to the domestic dog (B, D and F, respectively).

To study the anatomy of the orbit, heads of three crab-eating foxes and four domestic dogs were used. One crab-eating fox head was macerated, and the others were preserved from the topographic anatomical study of the eye and adnexa. According to the methodology described by Sarma [16], the following measurements were performed with the use of a digital caliper (Fig 1):

- Orbital vertical length: the perpendicular distance between the supraorbital and infraorbital margins of the orbit
- Orbital horizontal width: the horizontal distance between the rostral and caudal margins of the orbital rim
- Orbital index: orbital width/orbital length x 100
- Orbital depth: distance between the optic foramen and center of the orbital rim
- Orbital area: 22/7 ab, where a and b are half the orbital length and width, respectively
- Interorbital distance (Fig 2):
  - At rostral level: distance between the junction of the frontolacrimal sutures on either side at the rostral margin of the orbit
  - At middle level: distance between the supraorbital borders of the orbit on either side
  - At caudal level: distance between the junctions of the zygomatic bone at the caudal margin of the orbit on either side
- Frontal length: distance from the tip of the zygomatic process of the frontal bone to the frontolacrimal sutures
- Lacrimal length: distance from frontolacrimal sutures to the junction between the lacrimal and zygomatic bones
- Malar length: distance from the junction between the lacrimal and zygomatic bones to the tip of the frontal process of the zygomatic bones.

### Processing and histological evaluation of the eye and adnexa

For histological evaluation, both eyes from the head used for anatomical description of the orbit were processed. Fragments of all structures identified in the anatomical study were placed in cassettes for processing by routine histological paraffin-inclusion technique. Hematoxylin and eosin (HE) staining was performed on 4-μm sections. At the end of this stage, the histological sections were analyzed under an optical microscope with coupled camera (Leica Microscope, ICC50 E).

### Statistical analysis

The Shapiro–Wilk test was used to verify the normality of the variables obtained from measurements of the eye and adnexa. For analyses, the Prism Graphpad statistical program (version 7.04) was used and the level of significance was set at 5%. Comparison of the variables between left and right eye was performed using the Wilcoxon matched-pairs signed-rank test, and comparison between the two species was performed using the unpaired Mann–Whitney test.

## Results

Measurement data for the eye and adnexa were not normally distributed for either crab-eating fox (*p* < 0.019) or domestic dogs (*p* < 0.033) by Shapiro–Wilk test. In addition, no significant differences were found between the right and left eye for the variables studied in either species, according to the Wilcoxon test for paired samples (*p* > 0.125). In the CT study, images of the frozen head did not show good definition of soft tissue, with dark-colored structures and density similar to that of air.

In general, because CT imaging occurs by X-ray attenuation and due to the small size of periocular structures, evaluation (visualization and differentiation of structures) was not easy. Interspersion of adipose tissue among the structures facilitated the delimitation in several images.

### Adnexa

**Fig 3:**
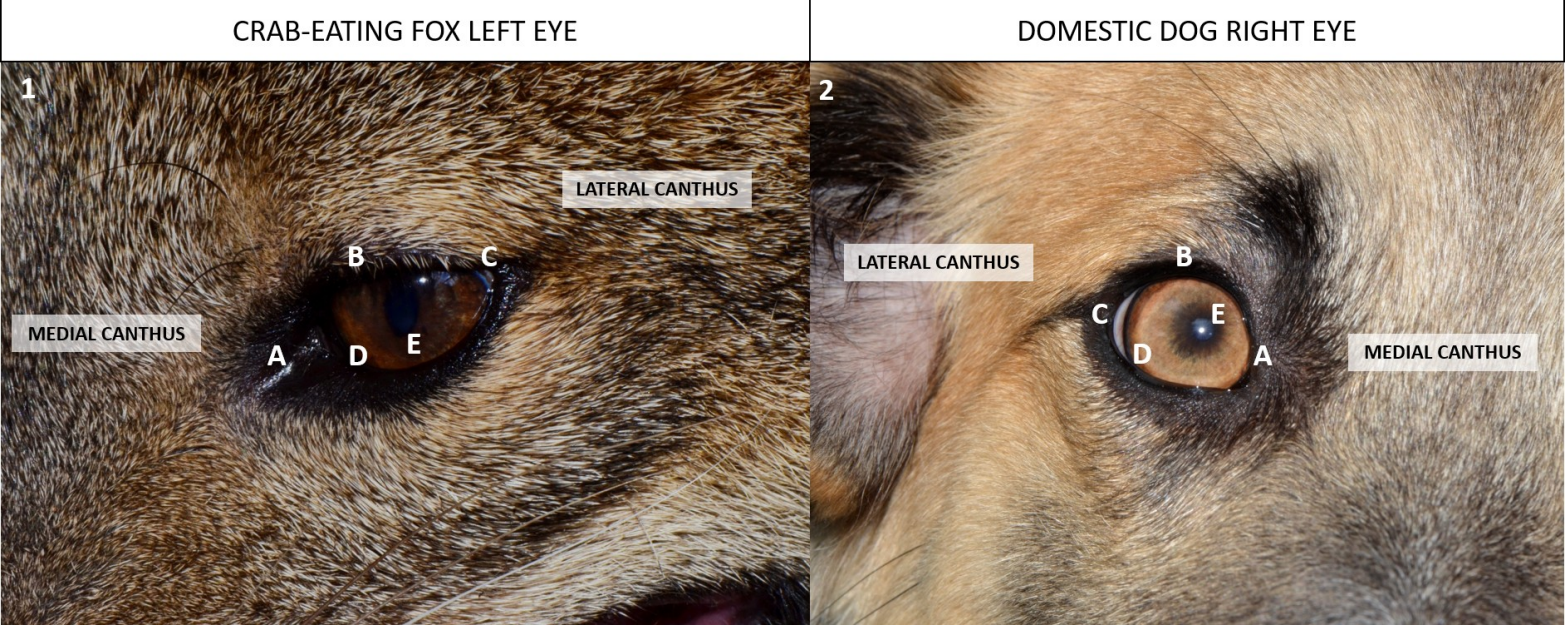
External view of crab-eating fox eye compared to canine eye. Completely pigmented upper and lower eyelids (A1 and A2), presence of cilia only on the upper eyelid (B1 and B2), minimal exposure of the slightly pigmented bulbar conjunctiva at the lateral canthus (C1 and C2), brown-colored iris (D1), and vertical subcircular-shaped pupil (E1). There are different colors of iris in dogs (D2) and a round pupil is observed (E2).

The upper and lower eyelids were completely pigmented in all animals, with cilia present only on the upper eyelid (Fig 3). Upper and lower lacrimal points were observed and their positions, along with palpebral fissure length measurements, are given in Table 1. There was no significant difference for palpebral fissure length between species (*p* = 0.065). However, there was a significant difference for the measured upper and lower lacrimal point distances between species (*p* < 0.0154), except the distance to the extremity of the lower tarsus (*p* = 0.1945).

**Table 1:** Palpebral fissure length and lacrimal point distances to the tarsal plate in crab-eating fox (n = 8 eyes) compared to the domestic dog (n = 10 eyes).

Variables	Crab-eating fox (mm)			Dog (mm)		
	Median	S-IQR	CI95%	Median	S-IQR	CI95%
**Palpebral fissure length**	17.32	1.49	16.51–19.47	21.32	3.47	18.33–24.31
Upper lacrimal point						
Tarsal distance	0.74	0.47	0.15–1.01	1.25	0.25	0.96–1,49
Tarsal extremity distance	0.01	0.33	-0.16–0.69	1.55	0.27	1.33–1.75
Lower lacrimal point						
Tarsal distance	0.92	0.36	0.33–1.04	1.32	0.20	1.16–1.49
Tarsal extremity distance	1.62	0.35	1.34–1.95	1.29	0.56	0.70–1.74

S-IQR, semi-interquartile range; CI95%, 95% confidence interval.

**Fig 4:**
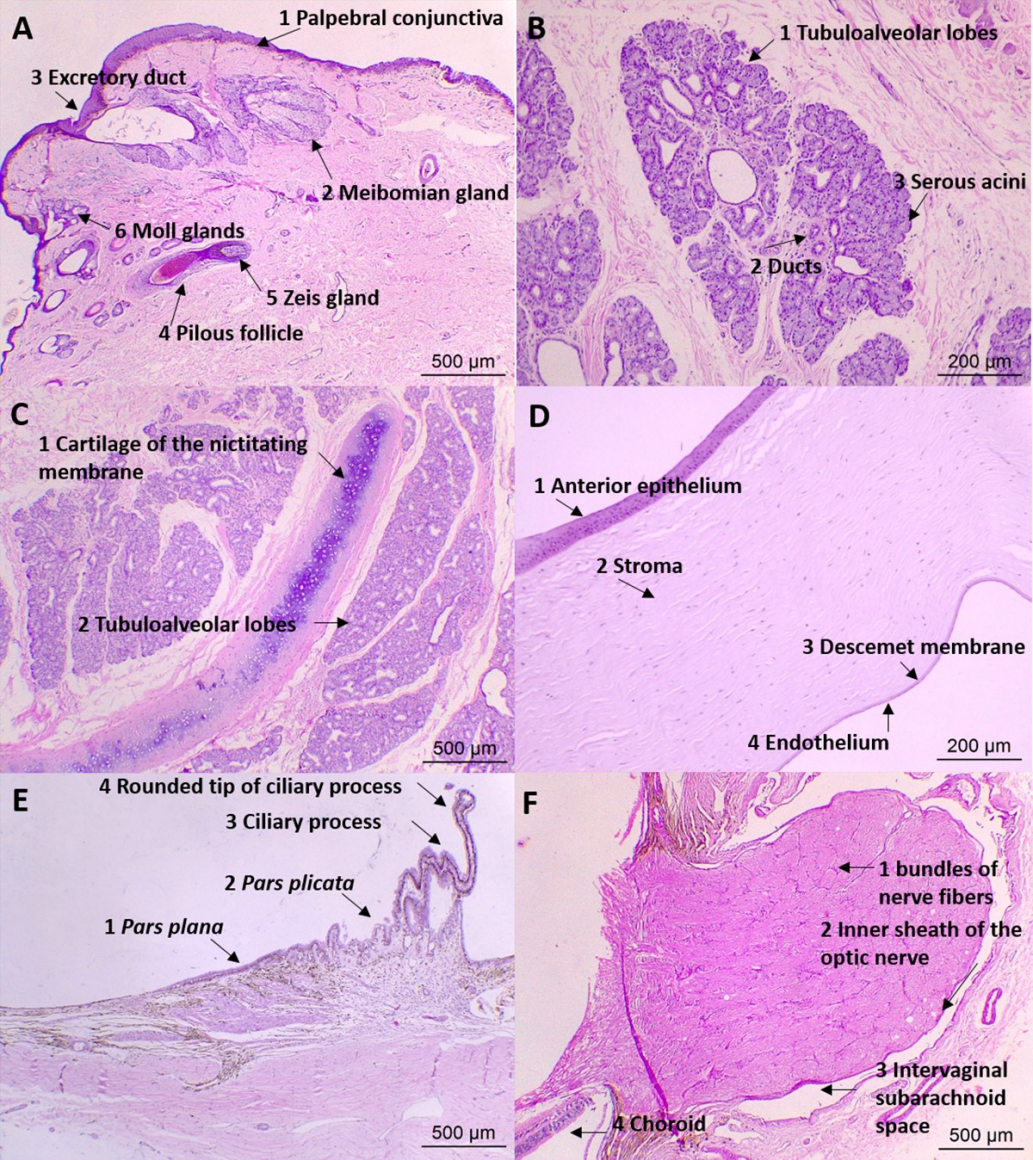
Photomicrography of the eye and adnexa of crab-eating fox. (A) Lower eyelid: 1, palpebral conjunctiva; 2, meibomian gland, with the presence of 3, excretory duct; 4, pilous follicle in close association with 5, the Zeis gland at its base, and 6, Moll glands; HE, 5X magnification. (B) Tubuloalveolar lacrimal gland formed by: 1, lobes and lobules; 2, ducts; 3, serous acini; HE, 10X magnification. (C) Nictitating membrane gland: 1, cartilage of the nictitating membrane; 2, tubuloalveolar lobes and lobules; HE, 5X magnification. (D) Cornea and its layers: 1, anterior epithelium; 2, stroma; 3, Descemet membrane; 4, endothelium; HE, 10X magnification. (E) Ciliary body: 1, pars plana; 2, pars plicata; 3, ciliary process, with the outer pigmented layer and inner nonpigmented layer, 4, rounded tip and presence of blood vessels; HE, 5X magnification. (F) Optic nerve head and optic nerve: 1, bundles of nerve fibers; 2, inner sheath of the optic nerve; 3, intervaginal subarachnoid space; 4, choroid; HE, 5X magnification.

Histologically, eyelids could be divided in four parts (Fig 4). The orbicularis oculi muscle was more developed in the upper eyelid, and in the cilium roots, sebaceous gland of Zeis and ciliary glands of Moll could be identified. A stromal layer of connective tissue with a thickening of dense connective tissue at the end revealed the meibomian gland, which was the largest sebaceous gland in the distal portion of the tarsal plate, opening into the palpebral margin. This gland was more developed in the lower eyelids of crab-eating fox than of the domestic dog.

The conjunctival pigmentation was slightly brownish throughout its extension and only the temporal border of the bulbar conjunctiva was exposed in crab-eating fox. Histology revealed prismatic, cylindrical or columnar epithelium. The lamina propria was composed of smooth connective tissue and along the epithelial cells, there was a large amount of melanin granules and lymphatic cells. Sparse Goblet cells could be identified in the bulbar conjunctiva, as well as in the nictitating membrane conjunctiva, surrounded by pigment.

**Fig 5:**
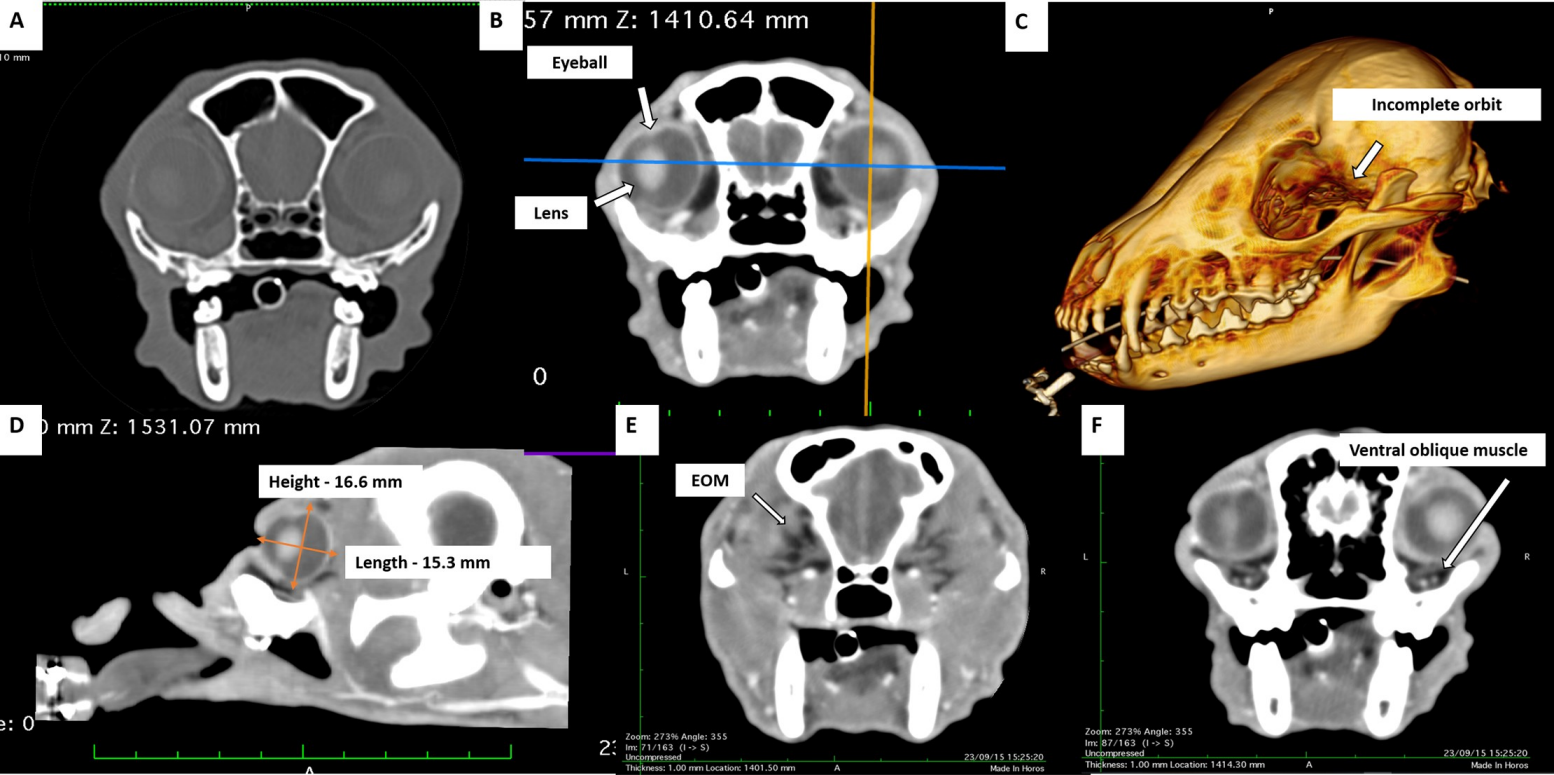
CT images of the eye and adnexa of crab-eating fox. Axial (transverse) plane image (A) without contrast, in bone window and (B) with contrast, in soft tissue window, where the eyeball contour, hypoattenuating internal content and hyperattenuating lens are evident. (C) Tridimensional bone reconstruction, with suppression of the soft tissues, showing the incomplete orbit (arrow). (D) Sagittal plane reconstruction image, in soft tissue window, showing the height and length of the eyeball (orange arrows). Contrast images in axial plane and soft tissue window (E) of the extraocular muscles (EOM) and (F) showing the pathway of the ventral oblique muscle.

The rectus dorsal muscle insertion on the dorsal surface of the eye was difficult to identify by CT with no contrast (Fig 5). It was just possible to follow its route to the optical foramen region. The oblique dorsal muscle was identified by the rectus dorsal muscle insertion on the eye, with no delimiting cleavage line between them, or by visualizing the insertion on the eye, ventrally to the rectus dorsal muscle. In this insertion, they seemed to be a single structure. The oblique dorsal muscle passed adjacent to the frontal bone, and then its route could no longer be observed. The trochlea could not be identified in either contrast or noncontrast CT images. The oblique dorsal muscle followed from the insertion dorsally on the eye to the frontal bone, juxtaposing it for a length of 7.0 mm, and could not be differentiated from the dorsal external ophthalmic vein and trochlea. Adipose tissue near these muscles was hypoattenuated compared to the muscle and its density varied from -28 to -84 HU.

Contrast CT images showed an elongated, tubular and symmetrical structure, with a visualized route from the region adjacent to the medial surface of the orbit (frontal bone) to the optic foramen (Fig 5). This tissue was only seen in the bone tissue window, slightly hyperattenuating relative to the soft tissue nearby and the bone. It was topographically compatible with the ophthalmic plexus.

On simple cross-sectional noncontrast and contrast CT of the topography of the medial rectus muscle insertion on the eye, a tissue with attenuation of soft tissue and indefinite contours was identified, extending to the surface of the frontal bone, without the possibility of isolating the medial rectus muscle or accompanying it in the caudal direction to the optic foramen.

In lateral rectus muscle topography, with contrast examination and transverse section, the lateral position of the eye showed an elongated structure with attenuation of soft tissues surrounded by hypoattenuating adipose tissue (-59 to -17 HU) which was directed to the optical foramen with an image that fused to other structures of the same attenuation in the bottom of the cone. In the noncontrast, cross-sectional examination, the same structure with attenuation from 22 to 46 HU was also identified.

In the simple CT examination, the ventral oblique muscle was ventrally identified on the eye and near the insertion of the ventral rectus muscle. The insertion of the ventral oblique muscle, with soft tissue attenuation, followed medially until insertion into the ventromedial face of the orbit, at the transition between the lacrimal and palatine bones. The insertion of the ventral rectus muscle was slightly hyperattenuating relative to the ventral oblique muscle. The muscle was directed caudally to the optic foramen, where its image fused with that of other muscles, and it was not possible to differentiate it. In the contrast examination, along the ventral rectus muscle course toward the optic foramen, a segment of the ophthalmic plexus juxtaposed to the muscle was identified.

In three sections, caudally on CT, ventral to the papilla, a discrete protuberance was observed, corresponding to the insertion of a group of retractor oculi muscle bundles. Both protuberances, in sequential cuts in the caudal direction, were shown as elongated structures directed toward the optical cone, where they appeared to merge with one another. It was not possible to isolate the other two bundles of the retractor oculi muscle. Among groups of muscle bundles, only fat attenuation tissue (-20 to -76 HU) was identified, with no possibility of identifying the optic nerve.

**Fig 6:**
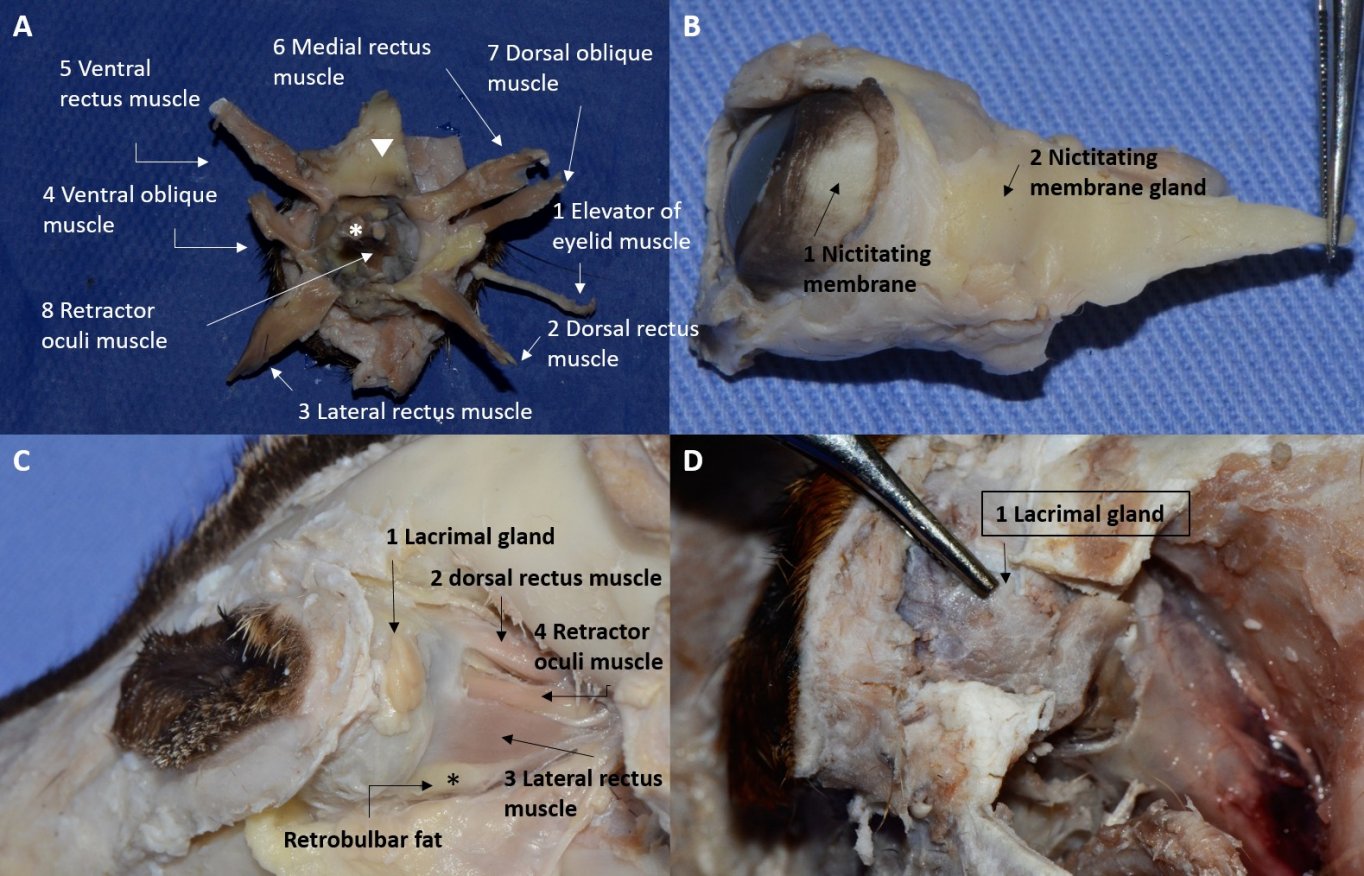
Gross anatomical image of the adnexa of crab-eating fox. (A) Posterior view of the extraocular muscles of crab-eating fox: 1, elevator of eyelid muscle; 2, dorsal rectus muscle; 3, lateral rectus muscle; 4, ventral oblique muscle; 5, ventral rectus muscle; 6, medial rectus muscle; 7, dorsal oblique muscle; 8, retractor oculi muscle. *Note the optic nerve and the inner face of the nictitating gland (arrowhead), covered by adipose and connective tissue. (B) Lateral view of the nictitating membrane of crab-eating fox: 1, palpebral face of the nictitating membrane showing pigmentation; 2, nictitating membrane gland covered by connective tissue. (C and D) Lateral view of the lacrimal gland showing macroscopic differences between crab-eating fox (C) and the domestic dog (D): 1, note the multilobulated oval shape in crab-eating fox and the irregular shape in the domestic dog; in the lateral aspect, it is possible to observe 2, the dorsal rectus muscle and 3, lateral rectus muscle, as well as 4, the retractor oculi muscle and *retrobulbar fat.

The following extraocular muscles (EOM) were significantly thinner in crab-eating foxes than in dogs (Table 2): dorsal rectus muscle, ventral rectus muscle, medial rectus muscle, dorsal oblique muscle and the retractor oculi (*p* < 0.0343). Their location is illustrated in Fig 6, and followed insertions similar to those described for dogs. A thinner trochlea was identified on the dorsal oblique muscle and the retractor oculi muscles could be divided into four muscle bundles surrounding the optic nerve and inserting posterior and deep into the rectus muscles. Yellowish adipose tissue covered the EOM, nictitating membrane gland (NMG) and optic nerve, and filled the dead retrobulbar space. Orbital fascia was identified covering the EOM and fat.

**Table 2:** Dimensions of the extraocular muscles (EOM) of the eye and eyelid elevator muscle in crab-eating fox (n = 8 eyes) and the domestic dog (n = 10 eyes).

**Eyelid elevator muscle**						
**Variables**	Crab-eating fox (mm)			Dog (mm)		
	Median	S-IQR	CI95%	Median	S-IQR	CI95%
**Length**	20.18	6.75	11.17–26.43	17.17	7.33	15.28–41.05
**Width**	2.74	0.36	2.36–4.10	3.31	0.69	2.60–4.49
**Thickness**	0.72	0.24	0.65–1.54	1.07	0.20	07.2–1.32
**Dorsal rectus muscle**						
**Variables**	Crab-eating fox (mm)			Dog (mm)		
	Median	S-IQR	CI95%	Median	S-IQR	CI95%
**Length**	20.97	2.32	15.76–26.58	28.47	4.18	24.49–40.30
**Width**	4.25	0.92	3.44–5.61	6.60	0.7175	5.78–8.07
**Thickness**	2.08	0.42	1.37–2.69	2.73	0.7175	2.15–3.63
**Ventral rectus muscle**						
**Variables**	Crab-eating fox (mm)			Dog (mm)		
	Median	S-IQR	CI95%	Median	S-IQR	CI95%
**Length**	21.43	4.18	18.03–28.82	25.17	5.96	20.32–35.72
**Width**	4.78	0.24	3.75–5.01	6.29	0.49	5.52–6.72
**Thickness**	2.50	0.27	1.95–2.74	3.06	0.50	2.30–3.63
**Lateral rectus muscle**						
**Variables**	Crab-eating fox (mm)			Dog (mm)		
	Median	S-IQR	CI95%	Median	S-IQR	CI95%
**Length**	20.68	2.30	17.71–24.80	26.95	6.90	18.34–35.59
**Width**	5.51	0.59	4.60–7.84	6.31	0.89	5.75–9.16
**Thickness**	2.45	0.34	1.90–2.67	2.56	0.50	2.14–3.70
**Medial rectus muscle**						
**Variables**	Crab-eating fox (mm)			Dog (mm)		
	Median	S-IQR	CI95%	Median	S-IQR	CI95%
**Length**	18.67	2.16	15.59–24.56	22.45	5.595	18.33–34.12
**Width**	4.77	0.5415	3.78–5.38	6.28	0.5835	5.51–6.94
**Thickness**	2.56	0.2865	2.18–2.97	3.31	0.6215	2.12–3.97
**Dorsal oblique muscle**						
**Variables**	Crab-eating fox (mm)			Dog (mm)		
	Median	S-IQR	CI95%	Median	S-IQR	CI95%
**Length**	20.88	1.125	18.69–25.39	25.97	7.855	22.10–42.78
**Width**	3.96	0.351	3.19–4.53	5.14	1.57	4.06–10.26
**Thickness**	1.21	0.2512	0.89–1.52	1.75	0.3615	1.47–2.95
**Ventral oblique muscle**						
**Variables**	Crab-eating fox (mm)			Dog (mm)		
	Median	S-IQR	CI95%	Median	S-IQR	CI95%
**Length**	15.37	2.465	11.58–21.71	17.57	3.14	15.18–22.56
**Width**	5.55	0.706	4.36–6.98	6.02	1.52	4.77–7.87
**Thickness**	1.83	0.171	1.47–2.11	1.95	0.7465	1.16–2.99
**Retractor oculi muscle 1**						
**Variables**	Crab-eating fox (mm)			Dog (mm)		
	Median	S-IQR	CI95%	Median	S-IQR	CI95%
**Length**	20.37	4.785	15.33–26.40	25.99	7.705	16.62–43.94
**Width**	5.20	1.084	2.20–6.08	6.27	1.651	3.18–7.45
**Thickness**	1.07	0.2162	0.66–1.32	1.19	0.3675	0.68–1.79
**Retractor oculi muscle 2**						
**Variables**	Crab-eating fox (mm)			Dog (mm)		
	Median	S-IQR	CI95%	Median	S-IQR	CI95%
**Length**	21.90	3.225	15.76–29.75	26.66	6.595	17.78–42.12
**Width**	4.62	1.045	3.04–5.66	6.67	2.06	3.64–8.33
**Thickness**	1.09	0.3977	0.37–2.00	1.12	0.1775	0.87–1.42
**Retractor oculi muscle 3**						
**Variables**	Crab-eating fox (mm)			Dog (mm)		
	Median	S-IQR	CI95%	Median	S-IQR	CI95%
**Length**	18.64	1.145	15.64–24.16	30.67	8.75	18.88–43.35
**Width**	4.30	0.555	3.15–5.04	6.20	1.75	3.90–7.73
**Thickness**	1.44	0.279	0.79–1.83	1.16	0.3075	0.96–1.72
**Retractor oculi muscle 4**						
**Variables**	Crab-eating fox (mm)			Dog (mm)		
	Median	S-IQR	CI95%	Median	S-IQR	CI95%
**Length**	18.66	4.725	12.92–26.44	29.70	1.115	15.27–48.60
**Width**	3.72	1.3375	2.49–8.31	6.99	2.461	4.75–11.13
**Thickness**	1.31	0.25	0.95–1.71	1.11	0.235	0.87–1.64

S-IQR, semi-interquartile range; CI95%, 95% confidence interval.

**Fig 7:**
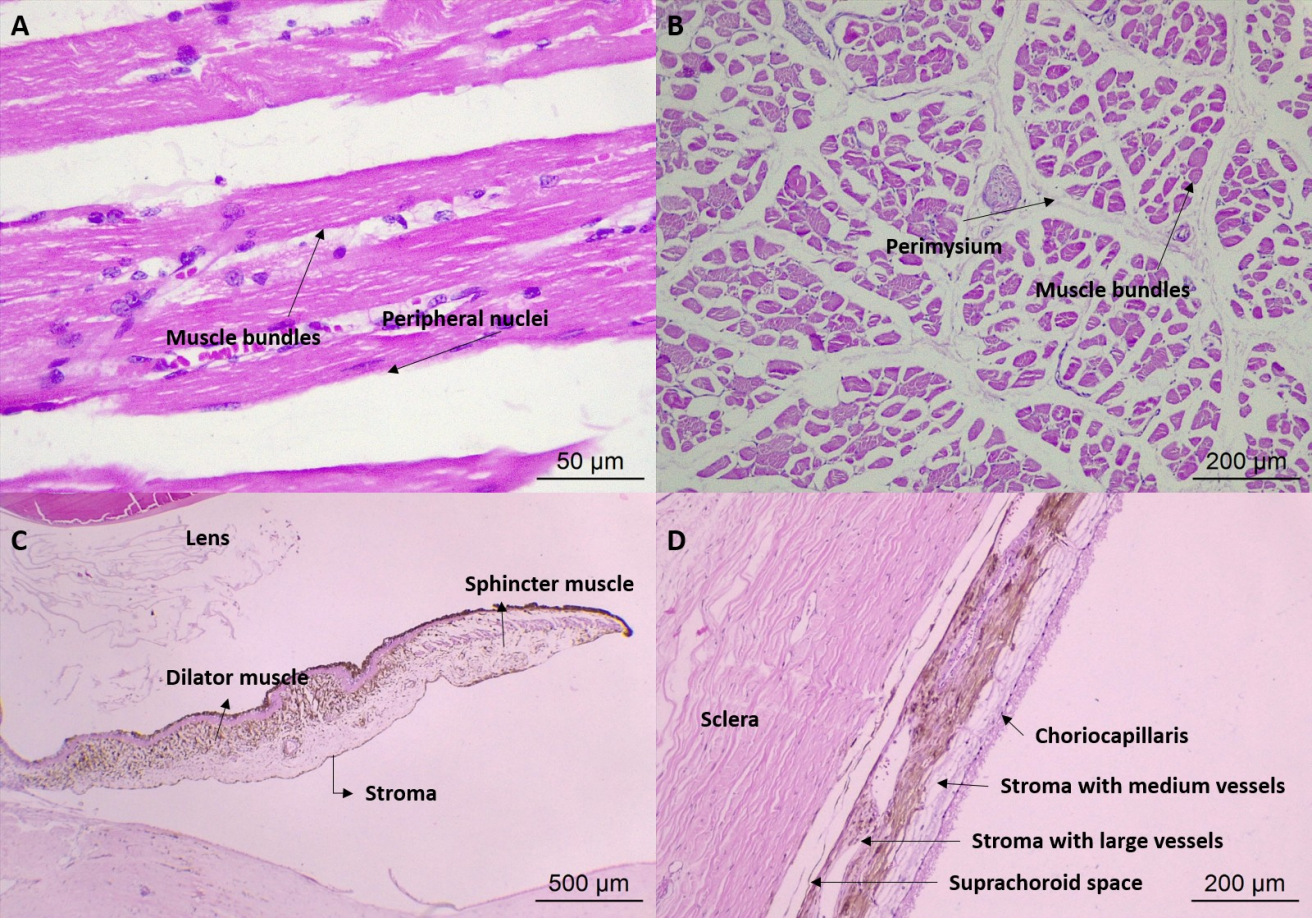
Photomicrography of the eye and adnexa of crab-eating fox. (A) Longitudinal section of an extraocular muscle: bundles of cylindrical fibers, elongated, multinucleated, with peripheral nucleus; HE, 20X magnification. (B) Transverse section of the extraocular muscle; HE, 10X magnification. (C) Iris: connective tissue stroma, the sphincter and dilator muscle; HE, 5X magnification. (D) Choroid: suprachoroid space, stroma with large vessels, stroma with medium vessels, and choriocapillaris; HE, 10X magnification.

In cross-sectional histological images, the striated skeletal musculature was composed of muscle fibers covered by endomysium and wrapped by perimysium, forming muscle bundles (Fig 7). These bundles were arranged in groups by the epimysium. In longitudinal sections, the transverse striations and bands A and I could be observed.

Immediately cranial to the eye and medially adjacent to the bone interface of the lacrimal and frontal bones, an attenuated soft tissue structure was identified, with poorly defined contours, which was topographically suggestive of the nictitating gland.

The nictitating membrane emerged from inferior medial canthus and its edge was pigmented (Fig 6). It contained a region of loose connective tissue around the gland and regions of dense connective tissue near the conjunctiva. It had a cartilaginous plaque inside and the seromucous NMG on the caudal portion. Measurement of the NMG included the lymphoid tissue and cartilage of the third eyelid. There was a significant difference in the length (*p* = 0.0031) of the nictitating membrane between the studied species. In addition, there was a significant difference between them in the total length (*p* = 0.0155), length (*p* = 0.0104) and width (*p* = 0.0043) of the NMG cartilage (Table 3).

**Table 3:** Dimensions of the nictitating membrane and nictitating membrane gland in crab-eating fox (n = 8 eyes) and the domestic dog (n = 10 eyes).

**Nictitating membrane**						
**Variables**	Crab-eating fox (mm)			Dog (mm)		
	Median	S-IQR	CI95%	Median	S-IQR	CI95%
**Length**	14.39	0.985	13.70–16.03	17.70	0.86	16.11–18.9
**Width**	6.48	0.5675	5.87–7.61	7.69	1.18	5.49–9.23
**Nictitating membrane gland**						
**Variables**	Crab-eating fox (mm)			Dog (mm)		
	Median	S-IQR	CI95%	Median	S-IQR	CI95%
**Total length**	24.50	2.53	21.98–28.99	19.30	2.81	15.75–23.80
**Gland length**	13.26	0.945	12.76–15.19	13.47	3.1575	8.07–15.67
**Width**	12.83	0.72	9.95–13.20	13.62	1.67	11.12–16.18
**Thickness**	4.57	1.7665	2.09–6.83	4.12	1.1675	3.48–6.40
**Cartilage length**	8.98	2.109	4.08–10.79	10.65	0.29	8.86–11.46
**Cartilage width**	5.52	0.605	4.40–7.10	7.44	0.9135	5.90–8.17

S-IQR, semi-interquartile range; CI95%, 95% confidence interval.

Visualization of the lacrimal gland (LG) was not easy by CT, and its contours were not very well defined. The supraorbital ligament facilitated its localization. The left eye gland had an oval aspect and was located, in cross-section on its largest axis, ventrally and adjacent to the ligament (Fig 5). There was a small amount of attenuating fat tissue between the ventral surface of the gland, the eye and the dorsal rectus muscle.

The right eye gland presented immediately caudal to the supraorbital ligament location. Part of the gland's image was fused to the dorsal rectus muscle and it was not possible to clearly delimit its contour. Visualization was only possible in cross-sectional images without contrast, because after the addition of contrast, the structures become hyperattenuating, making them impossible to differentiate in this region.

The LG was multilobulated, oval, nonencapsulated and flattened (Fig 6). It was located between the insertions of both the lateral and dorsal rectus muscle, under the supraorbital ligament, on the orbital edge, and was recognized after removal of the frontal muscle insertion, surrounded by intraocular fat. There was no significant difference between species (*p* > 0.0513) for the studied variables (Table 4). Histologically, the LG parenchyma was composed of a tubuloalveolar serous gland separated into lobes and lobules by connective tissue (Fig 4).

**Table 4:** Dimensions of the lacrimal gland in crab-eating fox (n = 8 eyes) and the domestic dog (n = 10 eyes).

Lacrimal gland						
Variables	Crab-eating fox (mm)			Dog (mm)		
	Median	S-IQR	CI95%	Median	S-IQR	CI95%
**Width (medial–lateral)**	9.18	5.52	7.44–19.28	14.2	2.69	8.82–15.19
**Length (anterior–posterior)**	7.13	2.76	3.66–10.72	8.50	1.02	7.55–14.72
**Maximum thickness**	3.30	1.36	1.93–6.68	3.28	1.10	2.43–4.83
**Minimum thickness**	1.59	0.78	1.28–3.72	1.56	0.49	0.82–2.17
**Distance to limbus**	6.45	1.07	5.87–8.48	9.17	2.14	6.59–13.72

S-IQR, semi-interquartile range; CI95%, 95% confidence interval.

The crab-eating fox orbit was incomplete, with the presence of a supraorbital ligament in the dorsolateral region. It had a triangular shape, with the apex facing the orbital edge. On CT, the supraorbital ligament was a thin softening attenuation structure extending from the surface of the frontal bone to the surface of the zygomatic bone, seen by about four to five 1-mm cuts in the cross-sectional areas (Fig 5).

The frontal, lacrimal, and zygomatic bones on the orbital edge, as well as the sphenoid, palatine and maxillary bones on the orbital floor were identified through the CT exam and confirmed by anatomical dissection. The rostral alar, optic and ethmoidal foramina, as well as the orbital fissure, were identified. The orbital measurements are shown in Table 5. There was a significant difference in orbital depth between the studied species (*p* = 0.0426). However, there was no significant difference between the interorbital distances in the two species (*p* > 0.0571). There was a significant difference in the length of the lacrimal bone between species according to the Mann–Whitney test (*p* = 0.0022).

**Table 5:** Orbital parameters measured for Crab-eating fox (n = 6 eyes) and the domestic dog (n = 8 eyes).

Orbital parameters						
Variables	Crab-eating fox (mm)			Dog (mm)		
	Median	S-IQR	CI95%	Median	S-IQR	CI95%
**Vertical length**	22.67	0.975	21.35–23.62	25.04	4.28	22.17–29.14
**Horizontal width**	19.41	3.20	16.72–22.83	21.97	2.94	18.97–25.18
**Orbital index**	91.08	12.40	100.7–75.2	97.54	17.49	107.50–71.96
**Orbital depth**	34.07	1.96	31.18–36.01	43.00	7.84	36.53–49.75
**Orbital area**	324.90	58.85	287.40–412.20	389.50	81.85	365.70–518.30
**Interorbital distance**						
At rostral level	25.21	1.06	22.50–27.80	29.80	6.10	20.55–41.39
At middle level	38.81	3.46	27.11–46.43	41.68	5.42	33.64–52.64
At caudal level	56.81	1.31	52.20–59.70	64.72	2.63	58.81–68.41
**Length of the frontal**	16.75	1.33	15.04–18.60	17.33	2.05	15.31–19.59
**Length of the lacrimal**	10.74	0.98	10.09–12.57	20.04	1.40	17.92–20.89
**Length of the zigomatic**	16.27	5.77	12.03–24.96	23.55	4.47	18.66–29.49

S-IQR, semi-interquartile range; CI95%, 95% confidence interval.

The orbital bones were hyperattenuating on the CT exam, and well defined and delimited using bone window evaluation (Fig 5). There was only a thin lamina of maxilla bone as an interface between the roots of the molar teeth and the periorbitae.

### Eye

In the precontrast CT images (Fig 5), the eye presented as a single layer of spherical tissue, with hypoattenuating internal content (-18 to 25 HU), in which it was not possible to identify the cleavage line among sclera, choroid and retina layers (three layers from 0.5 to 1.0 mm thickness). The sclera was grayish, composed of bundles of connective tissue rich in collagen fibers, parallel to the eye surface, covered by the bulbar conjunctiva and the limbus, which had a slight brown pigmentation. Episclera with more caliber vessels and supra choroidal lamina were observed. The transparent cornea was identified, with anterior chamber similar to that observed in dogs. Histologically, the cornea was divided into four layers: the anterior epithelium, with three to seven layers of nonkeratinized stratified squamous epithelial cells, thinner in the center and thicker near the limbus; stroma, with multiple layers of connective tissue; Descemet membrane; and posterior endothelium, a single layer of simple squamous epithelial cells (Fig 4).

Internally, CT images only differentiated the hyperattenuating lens, which thickened gradually in the centripetal direction. The lens had a biconcave shape with a larger posterior pole. However, the contour of the lens was smoky. In the postcontrast exam, a narrow tissue (26 to 50 HU) positioned between the dorsomedial and ventrolateral edges of the lens and the eye was identified, suggesting the ciliary body and iris, with no possible differentiation.

A brownish iris with central slit-shaped pupil was also recognized. Microscopically, this was made up of connective tissue stroma, the sphincter and dilator muscle (Fig 7). The ciliary body was identified, with the ciliary muscle and the ciliary processes attached and facing the posterior chamber (Fig 4). The lens was elliptical, smaller and more rounded than in dogs, and was attached to the ciliary body by zonular fibers on the lens pole. It consisted of three predominantly eosinophilic layers: lens capsule, subcapsular epithelium and lens fibers.

Four cuts before finishing the noncontrast cross-sectioned images of the caudal part of the eye, a region of contour discontinuity was visualized, suggestive of treating the optic papilla, with no possibility of identifying the optic nerve or adjacent blood vessels. In the adjacent region, hypoattenuating tissue (fat attenuation) was visualized, with a density ranging from -20 to -80 HU. Following a caudal cut, a discrete protuberance of soft part attenuation (14 to 25 HU) was already visualized, dorsal to the aforementioned region of the optic papilla. In cross-section contrast images, two vascular segments, one more dorsal and one more ventral, were identified adjacent to the optic papilla as a function of contrast filling, which delimited an attenuating tissue between soft tissues (Fig 5).

**Fig 8:**
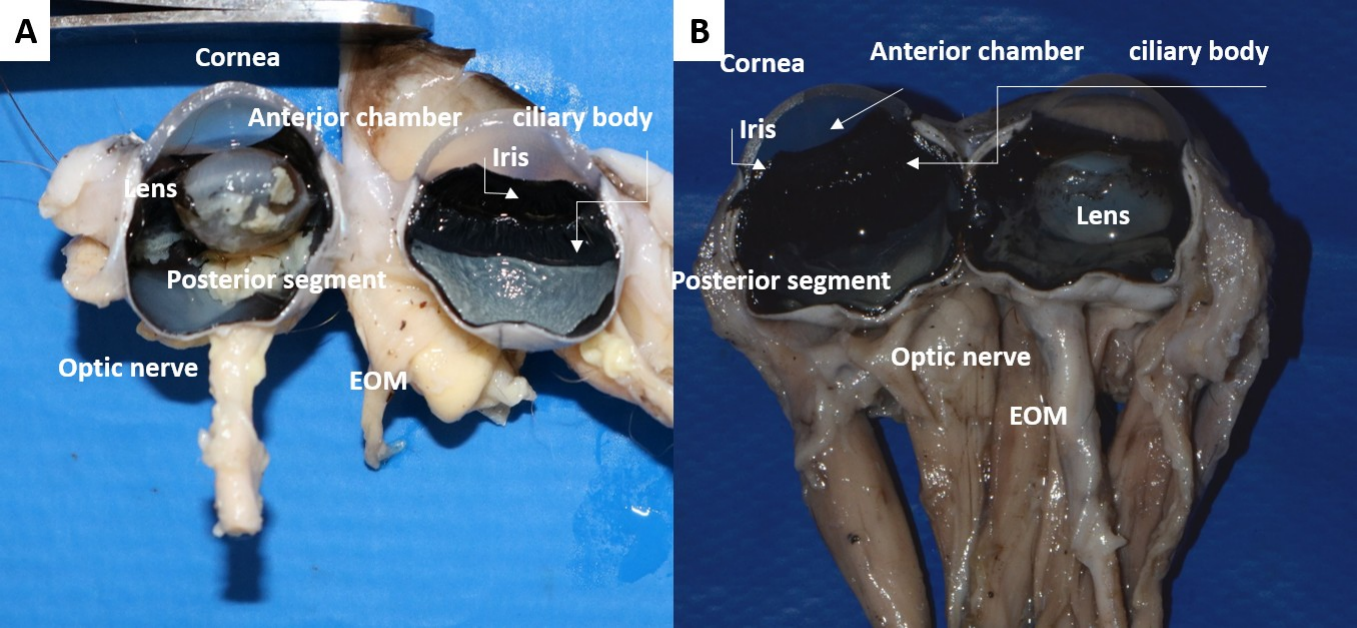
Internal structures of the eye fixed in formalin, in sagittal cut. (A) Crab-eating fox and (B) the domestic dog. Cornea, anterior chamber, iris and ciliary body, (d) lens, (e) posterior segment, (f) optic nerve, (g) extraocular muscles (EOM).

The tapetum lucidum was green in all animals (Fig 8) and the choroid appeared darkened. The choroid was limited anteriorly by the ciliary body and posteriorly by the margin of the optic nerve, between sclera and layers of retina. It was divided externally to internally into the suprachoroid space, stroma with large vessels, stroma with medium vessels (where the tapetum is located) and choriocapillaris (Fig 7). In the optic nerve region, some structures could be observed, such as bundles of nerve fibers, subarachnoid intervaginal space, outer sheath of the optic nerve, ciliary arteries and nerves, and the central retina artery (Fig 4).

Measurements for the eye are presented in Tables 6 and 7. The results of horizontal width (15.3–15.6mm) and vertical length (16.6–17.1mm) estimated by CT are within the confidence interval measured in formaline eyes (14.35–18.17 and 15.62–19.84, respectively). The vertical length and horizontal width of the fox’s eye were significantly smaller than in the domestic dog (*p* < 0.0155). However, there was no significant difference between corneal dimensions measured for both species (*p* > 0.1220). There was a significant difference in the thickness (*p* = 0.0266) of the optic nerve between species.

**Table 6:** Dimensions of eye in crab-eating fox (n = 8 eyes) and the domestic dog (n = 10 eyes).

Eye dimensions						
Variables	Crab-eating fox (mm)			Dog (mm)		
	Median	S-IQR	CI95%	Median	S-IQR	CI95%
**Vertical length**	16.25	0.38	15.62–19.84	19.41	1.495	17.17–21.19
**Horizontal width**	15.84	1.22	14.35–18.17	19.92	1.505	17.91–25.91
**Corneal dimensions**						
**Vertical length**	13.53	0.355	12.86–13.97	13.84	0.75	12.30–14.55
**Horizontal width**	13.52	0.435	12.69–14.28	14.32	0.89	12.30–15.64
**Optic nerve**						
**Preserved length**	15.61	2.695	12.66–21.12	16.44	4.05	12.66–24.42
**Thickness**	2.26	0.199	1.98–3.33	3.18	0.496	2.22–3.94

S-IQR, semi-interquartile range; CI95%, 95% confidence interval.

**Table 7:** Dimensions and attenuation degrees of the eye and adnexa of crab-eating fox by CT examination.

Structures	Dimensions (mm)	Attenuation (HU)
**Dorsal rectus muscle thickness**	1.3	-9–27
**Lateral rectus muscle thickness**	1.4	22–69
**Ventral rectus muscle**	0.9	19–41
**Medial rectus muscle**	-	39–76
**Dorsal oblique muscle thickness**	1.0	10–61
**Ventral oblique muscle thickness**	0.7	-2–9
**Ophthalmic plexus**	2.2–2.4	144–247
**Nictitating membrane gland**	2.8–3.5	45–63
**Lacrimal gland**	6.8 x 3.4	-
**Supraorbital ligament**	-	40–77
**Orbital distances (OD and OS)**		460–1200
** Eye–frontal bone (medial)**	3.0–3.3	-
Eye–frontal sinus (NC)*	2.6–5.5	-
Eye–frontal sinus (C)*	2.8–5.8/3.0–6.6	-
** Frontal–zygomatic bones**	21.0–22.0	-
** Middle distance**	23.0–24.0	-
** Caudal distance**	11.0–12.0	-
** Eye–Zygomatic bone (C)**	1.6–1.8	-
** Eye–Maxillary bone (C)**	6.3–7.2/5.0–7.5	-
** Eye–Palatine bone (C)**	5.0–8.8/ 5.0–8.3	-
** Eye–TMJ (C)**	21.8–25.0	-
**Orbital depth**	25.0–27.0	-
**Eye (OD and OS)**		-
** Mid-lateral axis**	15.3–15.6	-
** Dorsoventral axis**	16.6–17.1	-
**Lens (OD and OS)**		0.75–150
** Length**	8.2–8.5	-
** Width (anterior–posterior pole)**	6.2–6.3	-
**Optic papilla**	3.1–3.3	-

*The distance from the eye to the frontal bone, at the height of the frontal sinus, gradually increases from the most ventral portion to the dorsal portion.

HU, Hounsfield Unit; OD, oculus dexter (right eye); OS, oculus sinister (left eye); NC, non-contrast; C, contrast; TMJ, temporomandibular joint.

## Discussion

Structures similar to those already described for domestic dogs were found in crab-eating fox for all visual-system evaluation methods. CT allowed identification mainly of the adnexa; and a detailed study was only possible in the live animal, regardless of the presence of iodinated contrast. This is in agreement with Nascimento et al. [17], who reported no influence of iodinated contrast for the interpretation of *Sapajus* sp. CT images in the soft tissue window. In fact, there are varying reports on the use of contrast for wild species, either allowing better visualization or proving to be inadequate for identification of some structures in various reptilian species [18, 19].

Following a topographic approach, similar features of the eyelids, such as the pigmented tarsal plate, with cilia beginning on the medial quarter and extending to the lateral canthus of the upper eyelid, were observed for both species. The mean gross structures presented for crab-eating fox eyelids have been reported for other neotropical species, which occupies savannas and tropical forests [3], such as the capybara (*Hydrochaeris hydrochaeris*), which is continually exposed to high amounts of sunlight in their natural environment [15].

The influence of habitat was also noted by the brownish coloration of the bulbar conjunctiva of crab-eating foxes, also noted for *Sapajus* sp. and capybara eyes [15, 17], but present only in exposed areas of the canine conjunctiva, or in abnormal or chronic conditions [20–22]. Species inhabiting temperate forests, such as the American beaver, possess pigmentation limited to the limbal-based region, since those animals does not receive the same amount of sunrays in their natural environment, compared to neotropical species [15, 23]. The histological findings of the conjunctiva are in accordance with previous descriptions [24], and goblet cells were observed throughout the palpebral, bulbar and nictitating membrane conjunctiva of crab-eating foxes, as described for dogs, cats and other wild and laboratory animals [25–28].

The gross anatomical and CT evaluations revealed the same number and location of EOM as in domestic dogs [29]. During the CT examination, the same difficulties in evaluating insertions and trajectories of some EOM (rectum and dorsal oblique muscles) were encountered, with and without contrast. Visualization of these structures might be improved with the combined use of magnetic resonance imagery (MRI), due to its enhanced anatomical detail and soft tissue characterization [30].

Most of the EOM in the crab-eating fox were significantly thinner than those measured for the domestic dog. This may be related to the former's significantly shallower orbital depth. The presence and disposition of the orbital fat may also influence the conformation and positioning of the EOM [31], and in crab-eating fox, space-filling adipose tissue was observed among the rectus and oblique muscles, and the retractor oculi muscles and the optic nerve, forming the same cone noted in dogs [32].

The yellowish color of the retrobulbar fat, observed only in the crab-eating fox, could be accounted to the presence of lutein, beta-carotene and retinol, and lesser addition of unidentified carotenoids, as previously described for human yellow orbital fat [33]. The presence of the orbital fat was important for identifying some structures in the retrobulbar space on CT images; however, some EOM and the optic nerve could not be differentiated. It is probable that in the examination without contrast, the muscles, nerve and fat have very close attenuations, preventing their differentiation.

In the retrobulbar space, CT identified the NMG and LG of crab-eating fox but was limited in demonstrating their contours. This parameter is often required in assessing the lacrimal system, particularly for patients with medial canthus neoplasia, mid-face trauma or following surgery [34]. Gross anatomy and histology of the nictitating membrane, NMG and cartilaginous plaque were similar to dogs, with differences in the length and dimensions of the cartilage, perhaps related to a smaller proportion of crab-eating fox ocular structures. Therefore, the present imaging and morphological descriptions can be used as a comparative tool for diagnosis and therapeutic management in veterinary practice. However, further studies should be developed to better understand the lacrimal system, such as the use of tomographic dacryocystography, which has already been reported for dogs and cats [35, 36].

Even though the NMG and LG are important contributors to aqueous tear secretion and to maintaining ocular surface health, an anatomical description in dogs has only recently been reported [14]. As such, the present study can serve as an important tool for understanding lacrimal system functioning in crab-eating fox and for developing further studies into this system's biochemical components and physiology.

On precontrast CT images, the LG appeared in a consistent anatomical position underneath the supraorbital ligament, but with poor definition of its contours. These results differ from Zwingenberger et al. [37], who showed good visualization of the LG on postcontrast CT images of dogs. Those authors suggested that there is a weak correlation between body weight and LG volume, which could explain why the LG was not conspicuous for the crab-eating fox specimen.

In contrast to the domestic dogs studied, the crab-eating fox LG was observed even before supraorbital ligament removal, probably due to the proportions of the ligament. Its oval shape was similar to that reported for dogs, as well as bison and goats [14, 38, 39]. The dimensions of the LG on CT exam for crab-eating fox were slightly smaller than those described for domestic dogs [37]; however, the gross dimensions of LG and NMG for both species were similar to those reported for dogs of different breeds, with variations explained by breed or sexual dimorphism [14, 40, 41].

The gross anatomy and CT images described the crab-eating fox orbit as incomplete and composed of the same bony structures as in the domestic dog. Reports of crab-eating fox cranial morphometry have shown that variations in anatomical composition can be related to climate conditions and geographical adaptations of different populations [42]. Therefore, in the present study, a slightly but significantly shallower orbit was observed in crab-eating fox compared to the domestic dog. This was previously reported by Schmitt and Wallace [43], who analyzed cranial morphological features of North American wild canids (*Canis lupus*, *Canis latrans*, *Canis rufus*) compared to domestic dogs (*Canis familiaris*). Cranial shape and size on a ventral and lateral aspect were compared among species and the domestic dog was found to have the largest orbital dimensions.

The canine orbital expansion gives them the unique appearance of large eyes, a characteristic that has been artificially selected by humans. These variations are presumably due to the selective pressures experienced by domesticated breeds in the last decades and will probably continue to distinguish them even more saliently from their wild ancestors [43]. Therefore, the results observed in the present study could be explained by the different sizes of domestic dog–which possess eye’s dimensions ranging from 19.92 mm (present study) to 21.73 mm in the meridional axis, and 19.41 mm (present study) to 21.34 mm in the equatorial axis [44, 45]–and crab-eating fox eyes.

Crab-eating foxes have a significantly smaller eyeball (15.84 mm in the meridional axis and 16.25 mm in equatorial axis), with a cornea of the same size and shape as domestic dogs, suggesting that the cornea is proportionally bigger in the former. The pigmented conjunctiva, brownish iris, vertical slit-shaped pupil and more rounded lens might also be adaptations to the habitat and hunting behavior of the crab-eating fox [4, 46].

Banks et al. [47] evaluated animal pupil shapes and grouped them according to foraging habits, observing that most herbivores (prey) present a horizontally slit pupil whereas nocturnal or polyphasic ambush predators have a vertical pupil, and most daytime predators have circular pupils. This would explain the differences found between the two species in the present study, as the crab-eating fox has crepuscular habits and different feeding behavior relative to the domestic dog.

In addition, Malmström and Kröger [48] studied lens type and its relation to pupil format in some vertebrates, among them the domestic cat (*Felis silvestris domestica*) and the red fox (*Vulpes vulpes*), with vertical slit pupils; and the Siberian tiger (*Panthera tigris altaica*), the gray wolf (*Canis lupus lupus*) and the domestic dog (*Canis lupus familiaris*), with circular pupils. They authors suggested that the slit pupil seem to be a tool for adaptation to multifocal optical systems, i. e. for nocturnal and crepuscular vertebrates, in order to reach maximum light-gathering ability. However, the empirical measurement of the optical system status of the crab-eating fox needs to be investigated, to better understand its relation with lens optics and pupil shape.

## Conclusion

Given the presence of ocular structures similar to domestic dogs, the detailed CT and morphological descriptions presented in this study provides a useful foundation for veterinarians who work with the crab-eating fox. This may allow safer handling for diagnosis and treatment of ocular abnormalities and serve as a tool for conservation of the species, by recognizing ocular signs associated with systemic diseases, because the eye can be readily examined if the professional know the ocular morphology. In addition, considering the phylogenetic proximity with the crab-eating fox with other endangered canid species, it is possible that the results found in the present study will be useful for management of animals kept in captivity to minimize the impact of those diseases and allow the reintroduction of some populations.

The observed particularities of this wild species, such as the vertical slit-shaped pupil, presence of pigment throughout the bulbar conjunctiva and differences in some orbital and eyeball dimensions, reinforce the need for specific studies that will investigate the physiology and pathology of the visual system of crab-eating fox in more detail.

## Supporting information

S1 File SISBIO. Authorization and Information System of Biodiversity, Brazilian Ministry of the Environment–SISBIO (process no. 27489–1).
(PDF)

S2 File CEUA. Ethics Committee for the Use of Experimental Animals of the School of Veterinary Medicine and Zootechny, Federal University of Bahia (protocol no. 73/2016).
(PDF)
