# Supplementary material for: Anatomical, histological and computed tomography comparisons of the eye and adnexa of crab-eating fox (Cerdocyon thous) to domestic dogs
Source: PLoS One. 2019 Oct 23;14(10):e0224245. doi: 10.1371/journal.pone.0224245 (PMC6808443; doi:10.1371/journal.pone.0224245)
Supplement: S1 File — Authorization and Information System of Biodiversity, Brazilian Ministry of the Environment–SISBIO (process no. 27489–1). (PDF) [file pone.0224245.s001.pdf]

Ministério do Meio Ambiente - MMA
Instituto Chico Mendes de Conservação da Biodiversidade - ICMBio
Sistema de Autorização e Informação em Biodiversidade - SISBIO

Autorização para atividades com finalidade científica

Número: 27489-4	Data da Emissão: 06/01/2017 10:37	Data para Revalidação*: 05/02/2018

* De acordo com o art. 28 da IN 03/2014, esta autorização tem prazo de validade equivalente ao previsto no cronograma de atividades do projeto, mas deverá ser revalidada anualmente mediante a apresentação do relatório de atividades a ser enviado por meio do Sisbio no prazo de até 30 dias a contar da data do aniversário de sua emissão.

Dados do titular

Nome: Arianne Pontes Oriá	CPF: 645.585.505-10
Título do Projeto: Avaliação de Parâmetros Oftálmicos em Animais Silvestres	
Nome da Instituição : UNIVERSIDADE FEDERAL DA BAHIA	CNPJ: 15.180.714/0001-04

SISBIO

Cronograma de atividades

#	Descrição da atividade	Início (mês/ano)	Fim (mês/ano)
1	Avaliação de Parametros Oftálmicos em Animais Silvestres	03/2011	03/2013
2	Avaliação de Parametros Oftálmicos em Animais Silvestres	08/2016	08/2020

Observações e ressalvas

1	As atividades de campo exercidas por pessoa natural ou jurídica estrangeira, em todo o território nacional, que impliquem o deslocamento de recursos humanos e materiais, tendo por objeto coletar dados, materiais, espécimes biológicos e minerais, peças integrantes da cultura nativa e cultura popular, presente e passada, obtidos por meio de recursos e técnicas que se destinem ao estudo, à difusão ou à pesquisa, estão sujeitas a autorização do Ministério de Ciência e Tecnologia.
2	Esta autorização NÃO exime o pesquisador titular e os membros de sua equipe da necessidade de obter as anuências previstas em outros instrumentos legais, bem como do consentimento do responsável pela área, pública ou privada, onde será realizada a atividade, inclusive do órgão gestor de terra indígena (FUNAI), da unidade de conservação estadual, distrital ou municipal, ou do proprietário, arrendatário, posseiro ou morador de área dentro dos limites de unidade de conservação federal cujo processo de regularização fundiária encontra-se em curso.
3	Este documento somente poderá ser utilizado para os fins previstos na Instrução Normativa ICMBio n° 03/2014 ou na Instrução Normativa ICMBio n° 10/2010, no que especifica esta Autorização, não podendo ser utilizado para fins comerciais, industriais ou esportivos. O material biológico coletado deverá ser utilizado para atividades científicas ou didáticas no âmbito do ensino superior.
4	A autorização para envio ao exterior de material biológico não consignado deverá ser requerida por meio do endereço eletrônico www.ibama.gov.br (Serviços on-line - Licença para importação ou exportação de flora e fauna - CITES e não CITES).
5	O titular de licença ou autorização e os membros da sua equipe deverão optar por métodos de coleta e instrumentos de captura direcionados, sempre que possível, ao grupo taxonômico de interesse, evitando a morte ou dano significativo a outros grupos; e empregar esforço de coleta ou captura que não comprometa a viabilidade de populações do grupo taxonômico de interesse em condição in situ.
6	O titular de autorização ou de licença permanente, assim como os membros de sua equipe, quando da violação da legislação vigente, ou quando da inadequação, omissão ou falsa descrição de informações relevantes que subsidiaram a expedição do ato, poderá, mediante decisão motivada, ter a autorização ou licença suspensa ou revogada pelo ICMBio, nos termos da legislação brasileira em vigor.
7	Este documento não dispensa o cumprimento da legislação que dispõe sobre acesso a componente do patrimônio genético existente no território nacional, na plataforma continental e na zona econômica exclusiva, ou ao conhecimento tradicional associado ao patrimônio genético, para fins de pesquisa científica, bioprospecção e desenvolvimento tecnológico. Veja maiores informações em www.mma.gov.br/cgen.
8	Em caso de pesquisa em UNIDADE DE CONSERVAÇÃO, o pesquisador titular desta autorização deverá contactar a administração da unidade a fim de CONFIRMAR AS DATAS das expedições, as condições para realização das coletas e de uso da infra-estrutura da unidade.
9	As atividades contempladas nesta autorização abrangem espécies brasileiras constante de listas oficiais (de abrangência nacional, estadual ou municipal) de espécies ameaçadas de extinção, sobreexplotadas ou ameaçadas de sobreexplotação.

Outras ressalvas

1	Nesta quarta (4ª)RENOVAÇÃO de autorização ICMBio/SISBio, foi incluída uma nova área de coleta de amostra biológica ex situ, a saber o criatório comercial de jacaré-do-papo-amarelo Mister Cayman, localizado no município de Maceió, Alagoas, de propriedade da Srª Maria Cristina Muradas Ruffo.
2	Para a colheita de sangue em aves vivas, recomenda-se o máximo de 1% do peso vivo do indivíduo. Se houver coletas recorrentes, não se deve ultrapassar 2% do peso vivo a cada 14 dias.

Equipe

#	Nome	Função	CPF	Doc. Identidade	Nacionalidade
1	Melissa Hanzen Pinna	colaborador	073.593.677-35	087377602 IFP-RJ	Brasileira
2	CATERINA MURAMOTO	Pesquisador/ Colaborador	260.550.488-33	21817696x ssp-SP	Brasileira
3	Nayone Lima Lantyer Cordeiro de Araujo	Colaborador/ Pesquisador	047.961.135-17	1196350629 SSP-BA	Brasileira
4	Deusdete Conceicao Gomes Junior	Colaborador/ Pesquisador	017.644.215-40	0956332862 SSP-BA	Brasileira
5	Vinícius Brito	Colaborador/Pesquisador	057.688.125-20	1310137935 SSP-BA	Brasileira

Este documento (Autorização para atividades com finalidade científica) foi expedido com base na Instrução Normativa nº 03/2014. Através do código de autenticação abaixo, qualquer cidadão poderá verificar a autenticidade ou regularidade deste documento, por meio da página do Sisbio/ICMBio na Internet (www.icmbio.gov.br/sisbio).

SISBIO

Código de autenticação: 52669883

Autorização para atividades com finalidade científica

Número: 27489-4	Data da Emissão: 06/01/2017 10:37	Data para Revalidação*: 05/02/2018
* De acordo com o art. 28 da IN 03/2014, esta autorização tem prazo de validade equivalente ao previsto no cronograma de atividades do projeto, mas deverá ser revalidada anualmente mediante a apresentação do relatório de atividades a ser enviado por meio do Sisbio no prazo de até 30 dias a contar da data do aniversário de sua emissão.		

Dados do titular

Nome: Arianne Pontes Oriá	CPF: 645.585.505-10
Título do Projeto: Avaliação de Parâmetros Oftálmicos em Animais Silvestres	
Nome da Instituição : UNIVERSIDADE FEDERAL DA BAHIA	CNPJ: 15.180.714/0001-04

SISBIO

6	MARIA MADALENA SOUZA OLIVEIRA	Colaborador/Pesquisador	025.497.035-45	1259094626 ssp-BA	Brasileira
7	Débora Passos Hinojosa Schaffer	Colaborador/Pesquisador	014.897.095-84	0892201614 SSP-BA	Brasileira
8	Ana Cláudia Santos Raposo	Colaborador/ Pesquisador	033.009.505-62	0974475017 SSP-BA	Brasileira
9	JOÃO VICTOR ROMANO VIEIRA	Colaborador/ Pesquisador	044.494.765-57	1194656552 SSP-BA	Brasileira
10	Emanoel Ferreira Martins Filho	Colaborador/Pesquisador	924.803.495-00	0653256728 ssp-BA	Brasileira
11	EUNICE SANTOS DE ANDRADE	Colaborador/ Pesquisador	781.872.225-49	0832938416 SSP-BA	Brasileira
12	Alessandra Estrela da Silva Lima	Colaborador/ Pesquisador	899.826.575-34	438370694 SSP-BA	Brasileira
13	Francisco de Assis Dorea Neto	Colaborador/ Pesquisador	614.218.805-63	0531577711 SSP-BA	Brasileira
14	Ricardo Wagner Dias Portela	Colaborador/Pesquisador	028.924.927-93	094091535 IFP-RJ	Brasileira

Locais onde as atividades de campo serão executadas

#	Município	UF	Descrição do local	Tipo
1	SALVADOR	BA	Parque Zoobotânico Getúlio Vargas	Fora de UC Federal
2	SALVADOR	BA	Centro de Triagem de Animais Silvestres	Fora de UC Federal
3		AL	Mister Cayman	Fora de UC Federal

Atividades X Táxons

#	Atividade	Táxons
1	Coleta/transporte de amostras biológicas ex situ	Falconiformes, Didelphis albiventris, Passeriformes, Psittaciformes, Strigiformes, Callithrichidae, Canidae, Cebidae, Felidae, Podocnemididae, Procyonidae, Testudinidae, Sapajus, Iguana iguana, Caiman latirostris, Trachemys scripta, Cervidae, Tamandua tetradactyla, Chelonoidis carbonaria, Mesoclemmys, Didelphis aurita, Chelonoidis denticulata, Trachemys dorbigni, Galliformes

Material e métodos

1	Amostras biológicas (Aves)	Sangue, Fragmento de tecido/órgão, Outras amostras biológicas(lagrima), Animal encontrado morto ou partes (carcaça)/osso/pele
2	Amostras biológicas (Carnívoros)	Fragmento de tecido/órgão, Sangue, Animal encontrado morto ou partes (carcaça)/osso/pele, Outras amostras biológicas(lagrima)
3	Amostras biológicas (Outros mamíferos)	Sangue, Outras amostras biológicas(lagrima), Animal encontrado morto ou partes (carcaça)/osso/pele, Fragmento de tecido/órgão
4	Amostras biológicas (Primatas)	Animal encontrado morto ou partes (carcaça)/osso/pele, Sangue, Fragmento de tecido/órgão, Outras amostras biológicas(lagrima)
5	Amostras biológicas (Répteis)	Sangue, Animal encontrado morto ou partes (carcaça)/osso/pele, Fragmento de tecido/órgão, Outras amostras biológicas(lagrima)
6	Amostras biológicas (Tamanduás)	Outras amostras biológicas(lagrima)
7	Método de captura/coleta (Aves)	Puçá
8	Método de captura/coleta (Carnívoros)	Outros métodos de captura/coleta(Puca)
9	Método de captura/coleta (Outros mamíferos)	Captura manual, Puçá
10	Método de captura/coleta (Primatas)	Puçá, Captura manual
11	Método de captura/coleta (Répteis)	Coleta manual, Puçá

Este documento (Autorização para atividades com finalidade científica) foi expedido com base na Instrução Normativa nº 03/2014. Através do código de autenticação abaixo, qualquer cidadão poderá verificar a autenticidade ou regularidade deste documento, por meio da página do Sisbio/ICMBio na Internet (www.icmbio.gov.br/sisbio).

SISBIO

Código de autenticação: 52669883

Autorização para atividades com finalidade científica

Número: 27489-4	Data da Emissão: 06/01/2017 10:37	Data para Revalidação*: 05/02/2018
* De acordo com o art. 28 da IN 03/2014, esta autorização tem prazo de validade equivalente ao previsto no cronograma de atividades do projeto, mas deverá ser revalidada anualmente mediante a apresentação do relatório de atividades a ser enviado por meio do Sisbio no prazo de até 30 dias a contar da data do aniversário de sua emissão.		

Dados do titular

Nome: Arianne Pontes Oriá	CPF: 645.585.505-10
Título do Projeto: Avaliação de Parâmetros Oftálmicos em Animais Silvestres	
Nome da Instituição : UNIVERSIDADE FEDERAL DA BAHIA	CNPJ: 15.180.714/0001-04

SISBIO

12	Método de captura/coleta (Tamanduás)	Captura manual

Destino do material biológico coletado

#	Nome local destino	Tipo Destino
1	UNIVERSIDADE FEDERAL DA BAHIA	Ensino e pesquisa

SISBIO

Ministério do Meio Ambiente - MMA
Instituto Chico Mendes de Conservação da Biodiversidade - ICMBio
Sistema de Autorização e Informação em Biodiversidade - SISBIO

Autorização para atividades com finalidade científica

Número: 27489-4	Data da Emissão: 06/01/2017 10:37	Data para Revalidação*: 05/02/2018

* De acordo com o art. 28 da IN 03/2014, esta autorização tem prazo de validade equivalente ao previsto no cronograma de atividades do projeto, mas deverá ser revalidada anualmente mediante a apresentação do relatório de atividades a ser enviado por meio do Sisbio no prazo de até 30 dias a contar da data do aniversário de sua emissão.

Dados do titular

Nome: Arianne Pontes Oriá	CPF: 645.585.505-10
Título do Projeto: Avaliação de Parâmetros Oftálmicos em Animais Silvestres	
Nome da Instituição : UNIVERSIDADE FEDERAL DA BAHIA	CNPJ: 15.180.714/0001-04

SISBIO

Registro de coleta imprevista de material biológico

De acordo com a Instrução Normativa nº 03/2014, a coleta imprevista de material biológico ou de substrato não contemplado na autorização ou na licença permanente deverá ser anotada na mesma, em campo específico, por ocasião da coleta, devendo esta coleta imprevista ser comunicada por meio do relatório de atividades. O transporte do material biológico ou do substrato deverá ser acompanhado da autorização ou da licença permanente com a devida anotação. O material biológico coletado de forma imprevista, deverá ser destinado à instituição científica e, depositado, preferencialmente, em coleção biológica científica registrada no Cadastro Nacional de Coleções Biológicas (CCBIO).

Táxon*	Qtde.	Tipo de amostra	Qtde.	Data

* Identificar o espécime no nível taxonômico possível.

Este documento (Autorização para atividades com finalidade científica) foi expedido com base na Instrução Normativa nº 03/2014. Através do código de autenticação abaixo, qualquer cidadão poderá verificar a autenticidade ou regularidade deste documento, por meio da página do Sisbio/ICMBio na Internet (www.icmbio.gov.br/sisbio).

SISBIO

Código de autenticação: 52669883
