# Supplementary material for: Anatomical, histological and computed tomography comparisons of the eye and adnexa of crab-eating fox (Cerdocyon thous) to domestic dogs
Source: PLoS One. 2019 Oct 23;14(10):e0224245. doi: 10.1371/journal.pone.0224245 (PMC6808443; doi:10.1371/journal.pone.0224245)
Supplement: S2 File — Ethics Committee for the Use of Experimental Animals of the School of Veterinary Medicine and Zootechny, Federal University of Bahia (protocol no. 73/2016). (PDF) [file pone.0224245.s002.pdf]

Universidade Federal da Bahia
Escola de Medicina Veterinária e Zootecnia
Comissão de Ética no Uso de Animais
Av. Ademar de Barros, 500 – Ondina-40170-110 Salvador-BA
Fone: (071) 3283-6701/6702/6718
E-mail: escmev@ufba.br

CERTIFICADO

Certificamos que a proposta intitulada **"Avaliação de parâmetros oftálmicos, descrição anatômica, histológica e por tomografia computadorizada de olho e anexos em mamíferos silvestres"**, registrada com o nº **73/2016**, sob a responsabilidade da **Prof. Drª. Arianne Pontes Oriá**, e que envolve a produção, manutenção ou utilização de animais pertencentes ao filo Chordata, subfilo Vertebrata (exceto humanos), para fins de pesquisa científica (ou ensino), encontra-se de acordo com os preceitos da Lei nº 11.794, de 8 de outubro de 2008, do Decreto nº 6.899, de 15 de julho de 2009, e com as normas editadas pelo Conselho Nacional de Controle da Experimentação Animal (CONCEA), e foi aprovada pela COMISSÃO DE ÉTICA NO USO DE ANIMAIS (CEUA) da Escola de Medicina Veterinária da Universidade Federal da Bahia, **em reunião de 16.01.2017.**

Finalidade	( )Ensino (x) Pesquisa Científica
Vigência da autorização	16/03/2017 à 20/05/2020
Nº da solicitação ou autorização SISBIO	*27489-2*
Atividade(s)	☑ Captura: Contenção física<br>☐ Coleta de espécimes<br>☑ Coleta de material biológico: Secreção do saco conjuntival ventral e céculas da conjuntiva - Coleta com swab estéril para cultura com antibiograma e com escova interdental para citologia conjuntival.<br>☐ Marcação<br>☐ Outras:
Espécies/Grupos taxonômicos	Tamanduá-mirim, sariguê, cachorro-do-mato, lobo guará e caititu
Nº de animais	85
Sexo	Ambos
Origem	Centro de Triagem de animais Silvestres (CETAS – IBAMA)<br>Parque Zoobotânico Getúlio Vargas (PZBGV – Salvador)<br>Fazenda Gorgon – Criação Comercial de caititus – Irecê (BA)<br>Parque Fioravante Galvani – Luís Eduardo Magalhães (BA)
Local (is) de realização das atividades	Os animais serão mantidos em recinto próprio com água e alimentação disponíveis de acordo com as orientações do Centro de Triagem de Animais Silvestres, do Parque Zoobotânico Getúlio Vargas, da Fazenda Gorgon e do Parque Fioravante Galvani

Salvador, 16/01/2017.

Claudio de Oliveira Romão

Prof. Claudio de Oliveira Romão
Coordenador CEUA/EMEVZ-UFBA
